# Intra-articular venous malformations of the knee: a diagnostic challenge

**DOI:** 10.1186/s12969-021-00640-z

**Published:** 2021-10-13

**Authors:** Federico Diomeda, Maria Santaniello, Giulia Bracciolini, Angelo Ravelli, Adele Civino

**Affiliations:** 1 Dipartimento di Scienze Biomediche e Oncologia Umana, Università degli Studi di Bari Aldo Moro, Bari, Italy; 2 Dipartimento di Neuroscienze, Riabilitazione, Oftalmologia, Genetica e Scienze Materno-Infantili (DINOGMI), Università degli Studi di Genova, Genoa, Italy; 3 UOC Pediatria e Emergenza Pediatrica, Azienda Ospedaliera SS Antonio e Biagio e Cesare Arrigo, Alessandria, Italy; 4 Direzione Scientifica, IRCCS Istituto Giannina Gaslini, Genoa, Italy; 5 Sechenov First Moscow State Medical University, Moscow, Russia; 6 UOSD Reumatologia e Immunologia Pediatrica, Ospedale Vito Fazzi, Lecce, Italy

**Keywords:** Intra-articular venous malformation, Synovial hemangioma, Vascular malformation, Arthritis, Differential diagnosis

## Abstract

**Background:**

Intra-articular venous malformations (IAVM) are rare benign vascular anomalies that usually affect young patients and most common locate in the knee. The terminology of these lesions is still ill-defined, as they are often termed in the literature as synovial hemangiomas. Early diagnosis can be difficult, because they usually present with nonspecific clinical manifestations that are similar those of other rheumatic diseases, especially juvenile idiopathic arthritis (JIA).

**Case series:**

We conducted a retrospective analysis of five pediatric patients admitted to our units for recurrent swelling of the knee, and compared their characteristics with those of literature reports. The average age at first symptom and time from onset to diagnosis was 3.9 years (range 18 months-7 years) and 3.5 years (range 1-7 years), respectively. In our patients, an initial misdiagnosis of JIA, bleeding disorder or traumatic arthropathy was made. On MRI imaging, the features of the lesion were similar in all patients, and were marked by isointense-to-hypointense signal in T1-weighted images, and hyperintense signal in T2-weighted images. When performed, arthrocentesis led to aspiration of bloody fluid. The diagnosis was confirmed with a biopsy and histopathologic assessment in all patients. Open surgery enabled complete excision of the mass and was followed by stable remission over time in all cases.

**Conclusions:**

Our report highlights the challenges that may be posed by the detection of knee IAVM and the frequent long delay between onset of symptoms and diagnosis. The key elements for early recognition include careful assessment of patient history, demonstration of bloody fluid on arthrocentesis, and proper interpretation of MRI scanning.

## Background

Intra-articular venous malformations (IAVM) are rare benign vascular anomalies that usually affect young patients and most common locate in the knee [1–3]. The terminology of these lesions is still ill-defined, as they are widely termed in literature as synovial hemangiomas. However, nowadays they are labelled as IAVM in most reports.

Early diagnosis of these lesions can be difficult. When they present with the sudden onset of painful knee swelling after a local trauma, they may suggest a traumatic arthropathy or a congenital coagulation disorder. In case of insidious development, with painless swelling and no history of trauma, they can be misdiagnosed as juvenile idiopathic arthritis (JIA) [4] or as another inflammatory or non-inflammatory condition [1, 3]. As a result, several years frequently lapse between onset of symptoms and treatment [5].

In this report, we describe our experience with the diagnosis and treatment of IAVM in five patients, and highlight the role of magnetic resonance imaging (MRI) in their detection.

## Case reports

### Case 1

**Fig. 1:**
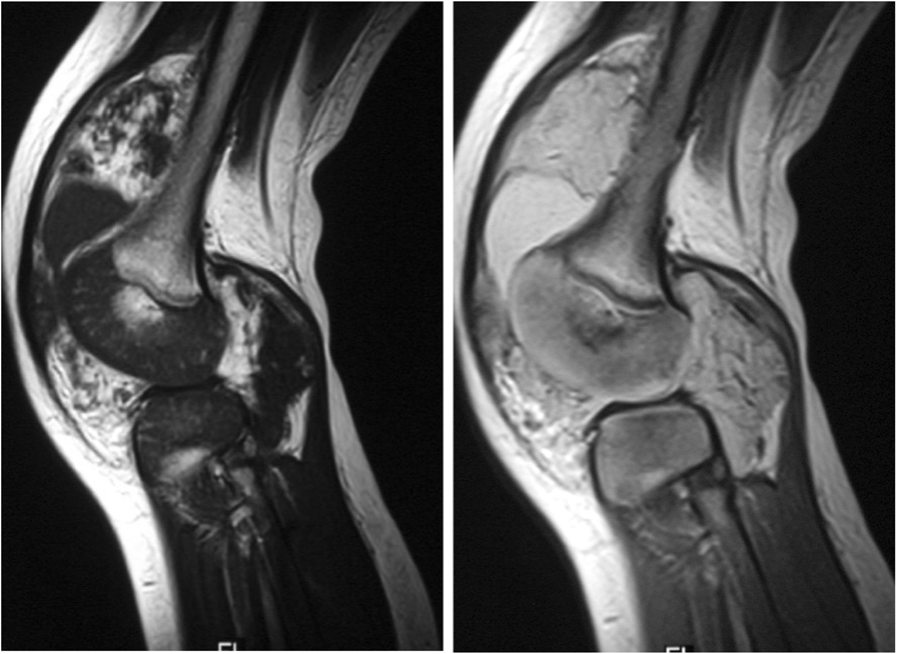
Knee MRI of patient 1. Sagittal T1 and T2 -weighted images show the extension of the IAVM in the synovial space

**Fig. 2:**
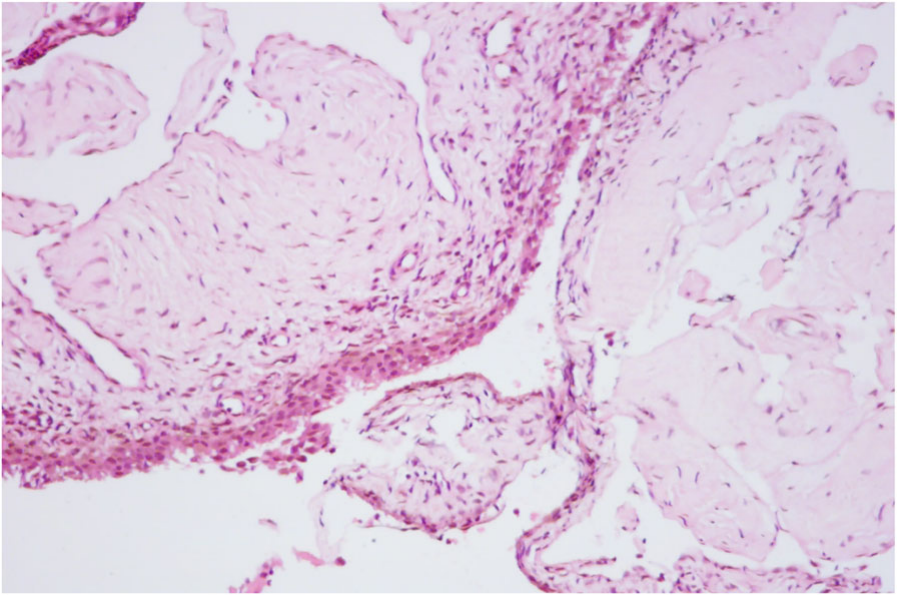
The histopathologic specimen of patient 1 shows that the lesion includes markedly dilated blood vessels

A 16-month-old boy developed a painless swelling of his right knee after a fall to the ground. He was brought to his local hospital, where a knee radiograph was performed, which did not disclose any abnormality. A diagnosis of traumatic arthropathy was made. A few days of weight-bearing avoidance and ice-pack application led to quick reduction of joint swelling, which, however, did not resolve completely. Indeed, over the following months the right knee always appeared to the parents slightly more swollen than the contralateral. Moreover, recurrent exacerbation of swelling, often following a minor trauma, was noted. One year after the onset of symptoms, the child was hospitalized elsewhere to investigate the cause of persistent right knee swelling. All laboratory tests, including acute phase reactants, antinuclear antibodies, and rheumatoid factor, and a further plain radiograph were negative. A diagnosis of JIA was made and nonsteroidal anti-inflammatory (NSAID) therapy with ibuprofen was prescribed, which had no appreciable effect. The boy was seen at the outpatient clinic of our unit at the age of 3 years, shortly after a further episode of knee swelling, preceded by a mild local trauma. On clinical examination, the right knee appeared swollen, but was modestly tender and painful on passive motion; considerable joint effusion and slight restriction of flexion were detectable. The remaining joints and the general physical findings were within normal limits. Knee ultrasonography revealed a bulk of hyperechogeneic material in the supra-patellar region, suggesting prominent synovial hypertrophy, and marked effusion. Since there was a discrepancy between the clinical and ultrasonographic findings potentially consistent with JIA and the history of a close relationship between the exacerbation of joint swelling and previous trauma, an arthrocentesis was performed. Aspiration led to evacuate 20 ml of bloody fluid. To rule out a bleeding disorder, the clotting tests were requested, which yielded normal results. An MRI of the knee was, then, performed, which demonstrated an extensive lesion, with low signal in T1-weighted images and high signal in T2-weigthed and stir images, nearly occupying the entire synovial cavity, and a large fluid collection (Fig. 1). A diagnostic arthroscopy led to the histopathological diagnosis of IAVM (Fig. 2). One week later, the lesion was resected surgically. At 3 months after surgery, the right knee did not show any evidence of swelling or fluid.

### Case 2

A 3-year-old girl presented to her family physician with a painful swelling of her left knee, which was attributed to a recent bump into a home furniture. The joint symptoms resolved within a few days with NSAIDs therapy. In the following years, she experienced intermittent episodes of swelling of her left knee, often preceded by a trauma. Six years after the onset of symptoms, an arthrocentesis of the affected knee revealed hemarthrosis. A bleeding disorder was sought and the coagulation tests showed decreased fibrinogen levels, which led to hypothesize a defect in fibrinogen synthesis. For this reason, the subsequent episodes of knee swelling were treated with an anti-fibrinolytic agent (tranexamic acid), which, however, did not improve symptoms. On admission at our hospital, 1 year later, the left knee appeared slightly swollen, but was not tender, and no effusion was detectable. An MRI showed a mass in the anteromedial part of the synovial cavity, which appeared slightly intense in T1-weighted and hyperintense in T2-weighted images. Diagnostic arthroscopy and biopsy revealed an IAVM. The mass was excised surgically. At 1-year follow-up, the girl was free of symptoms.

### Case 3

A 7-year-old boy was conducted to his local hospital because of the occurrence, in the absence of an identifiable previous trauma, of swelling, aching and functional limitation of his right knee. A knee radiograph was negative. A traumatic arthropathy was diagnosed. The knee was put in plaster and NSAIDs therapy was given for 1 month with remarkable improvement. However, 2 months later the swelling of the right knee recurred after a minor injury. Arthrocentesis led to aspiration of bloody fluid. The screening for coagulation defects was negative and the swelling regressed spontaneously within a few days. After 2 years without complaints, the boy had a new episode of painful knee swelling following a trauma. Repeated coagulation screening and a plain radiograph did not reveal abnormalities. When the boy was seen at our hospital, the right knee appeared slightly swollen, but neither painful nor functionally limited. An MRI revealed a mass in the retropatellar region, that was best demonstrated on T2-weighted images, without synovial effusion. Arthroscopy and biopsy confirmed the diagnosis of IAVM and the mass was removed surgically. During the 6-year follow-up, no recurrence of knee swelling was registered.

### Case 4

**Fig. 3:**
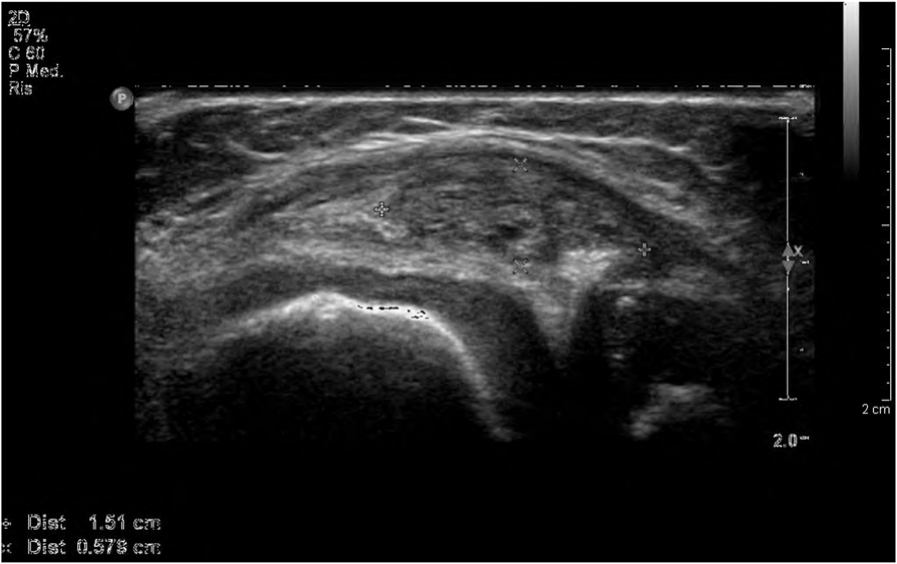
Ultrasonography of patient 4 shows a hyperechogenic mass in the medial part of the right knee

A 4-year-old girl was evaluated at the local hospital because of the occurrence of pain and functional limitation of her right knee, preceded by a minor trauma. She undergone laboratory tests as well as plain radiographs of the affected joint, which were all negative. A diagnosis of traumatic arthropathy was made and 1-week immobilization of the right leg and NSAIDs were prescribed, which led to satisfactory improvement. One year later, a new episode of painful right knee swelling occurred and two subsequent MRI of the affected joint were performed, which were not conclusive. When we first saw the girl, 8 months later, she had swelling and valgus deformity of the right knee. Ultrasonography revealed a hyperechogenic mass in the medial part of the right knee (Fig. 3). A further MRI revealed, on T2-weighted images, a heterogeneous hyperintense area with low contrast enhancement between the medial meniscus and collateral ligament. All relevant laboratory tests, including the coagulation profile, were within the normal range. The lesion was removed by open surgery and histologic evaluation confirmed the low-flow vascular nature.

### Case 5

A 4-year-old boy was conducted to his local hospital because of swelling, aching and functional limitation of his right knee after a trauma. A knee radiograph was negative and swelling resolved in 3 weeks. In the following years, he experienced intermittent episodes of swelling of right knee, without pain, often preceded by a trauma. At the age of 10 years, an MRI of the knee revealed joint effusion. All laboratory tests, including inflammation parameters, antinuclear antibodies and coagulation tests yielded normal results. No treatment was prescribed. On admission at our hospital, the right knee appeared slightly swollen, but neither painful nor functionally limited. The MRI scan of the affected knee revealed a mass occupying mainly the anterior compartment, with a high fat saturation signal, a low T1 signal, and a hyperintense T2 signal with high gadolinium enhancement. This mass appeared in close contact with the bone cortex of femoral condyles. Arthroscopy and biopsy confirmed the diagnosis of IAVM and the mass was removed surgically.

The main clinical features of the reported patients are summarized in Table 1.

**Table 1:** Main clinical features of reported patients

Age at symptoms onset/sex	Symptoms at first evaluation	Time to diagnosis	Arthrocentesis performed	Localization	History of trauma	Cutaneous signs of VM	Treatment	Recurrence
16 mo / M	Swelling.	18 mo	Yes, bloody	Knee, intrasynovial, extensive.	+	-	Surgical excision	-
3 yr / F	Pain, swelling.	7 yr	Yes, bloody	Knee, intrasynovial, anteromedial.	+	-	Surgical excision	-
7 yr / M	Pain, swelling, restricted joint motion.	2 yr	Yes, bloody	Knee, intrasynovial, retropatellar.	-	-	Surgical excision	-
4 yr / F	Pain, swelling, restricted joint motion.	1 yr	Not performed	Knee, intrasynovial, medial.	-	-	Surgical excision	-
4 yr / M	Pain, swelling, restricted joint motion.	6 yr	Not performed	Knee, intrasynovial, anterior.	+	-	Surgical excision	-

## Discussion and conclusions

Vascular anomalies are congenital abnormalities of vascular development. The International Society for the Study of Vascular Anomalies (ISSVA) [6] classified these conditions into vascular malformations and proliferative vascular lesions, like hemangiomas. Most hemangiomas are of the infantile type, which is characterized by excessive proliferation in the first year of life, followed by spontaneous subsequent regression. Vascular malformations are collections of enlarged aberrant and ecstatic vessels, with normal non-proliferative endothelium. They are present at birth and increase in size in parallel with child’s growth [7, 8].

In most literature reports, vascular abnormalities of the joints are named synovial hemangiomas, but in the majority of cases the microscopic features of these lesions are typical of a vascular venous malformation. In some studies, only intra-articular/intra-synovial lesions were comprised in the group of synovial hemangiomas [1], whereas other reports also included intra-articular/extra-synovial forms [2], which raises confusion in their classification. Mattila et al. [9] stated that they had never seen a real synovial hemangioma of the knee in their experience in tertiary referral center for vascular anomalies in children.

The knee is the most frequent reported location of IAVM, accounting for 60 % of all cases [1, 5]. In a large cohort of non-syndromic lower extremity IAVM, knee involvement was observed in 97/156 (62 %) patients [10]. IAVMs seem overall rare, as this lesion was found in only one of 4682 knee arthroscopies [11]. A variety of other joints can be affected, beside the knee, including the temporomandibular joint [12].

The typical patient with an IAVM is a child or young adult who presents with swollen and painful knee [1, 3]. Pain is always reported at the time of the first evaluation [2, 3, 13]. It can be acute, secondary to intra-lesional thrombosis or hemarthrosis, or chronic, secondary to chondropathy or muscle impairment [4]. The intra-lesional thrombosis of an IAVM is related to a local intravascular coagulopathy that can manifest as an increase in D-dimer blood level. This complication is more frequent in larger IAVM [3, 14, 15]. In our case 2 the low level of fibrinogen led the clinicians to misdiagnose of coagulation defect and to prescribe an erroneous treatment.

Another frequent symptom is limitation of the range of motion [2], that can be useful to differentiate isolated soft tissue vascular malformations from IAVM [4]. Cutaneous changes suggesting a vascular malformation, such as prominent veins or superficial venous dilation, can occasionally be observed in proximity of the affected joint, which helps to make an early diagnosis [3, 4, 16]. Occasionally, a tender spongy or firm mass may be palpable [1, 17]. In all our patients, the lesion was not recognizable clinically on physical examination.

Limb overgrowth is frequently observed in certain disorders with vascular malformations, as Klippel-Trénaunay syndrome [7]. In non-syndromic IAVM, the majority of the patients have either a normal limb length or a slight undergrowth or overgrowth of the affected extremity [16–18].

A previous trauma is reported in literature in a minority of cases of IAVMs [1, 2, 13]. Unlike these experiences, in our patients the episodes of knee pain and swelling were frequently heralded by a minor trauma. However, it is often difficult to establish reliably the cause-effect relationship with a trauma because children are exposed to frequent trauma during play and sport.

According to Moon et al., the average age of onset of IAVM is 10.9 years in girls and 12.5 years in boys, with the start of symptoms in 75 % of patients before the age of 16 [5], but no sex predilection can be defined [1, 5]. There has been a report in a 4-month-old boy [2]. The diagnosis of IAVM is often delayed, as preoperative diagnosis is difficult [5]. In our patients, the average age at first symptom was 3.9 years (range 18 months-7 years), and the average time to diagnosis was 3.1 years (range 1.5 – 7 years). This time lag is in the range of that reported in the literature, which varies from 2.2 to 6.7 years [2–4, 9, 19]. Late diagnosis is associated with worse prognosis after surgical treatment, with the possible development of structural joint changes leading to functional impairment [20], which may require additional orthopedic surgical treatment to restore mobility [15].

The course of IAVM is often chronic and is marked by alternance of relapses and remissions. Exacerbation of joint swelling is typically to intra-articular bleeding. In case of intra-synovial lesions, the synovia may become inflamed and hypertrophic, and progressive damage of cartilage and bone may ensue [1, 16, 21]. Intermittent reappearance of swelling with partial regression over time, as detected in all our cases, is a useful clinical feature to raise the diagnostic suspicion.

The differential diagnosis of IAVM include several causes of monoarthritis in childhood, including JIA, pigmented villonodular synovitis (PVNS), lipoma arborescens, juxta-articular mixoma, bursitis, malignant tumours [22], posttraumatic organizing hemorrhage, tuberculosis, sarcoidosis and bleeding disorders [1, 3]. Tuberculosis is often forgotten in the differential diagnosis of knee monoarthritis. It is good clinical practice to rule out tuberculous arthritis before diagnosing JIA. The presenting clinical features of monoarticular JIA can be very similar to those of IAVM, and both conditions may favorably to NSAID therapy in their initial stage. However, the aspiration of bloody fluid from the joint serves to distinguish these two disorders. Misdiagnoses in our patients included JIA, bleeding disorder, and traumatic arthropathy.

Imaging findings play a fundamental role in distinguishing IAVM from JIA and whenever possible, it is advisable to complete the diagnostic workup for persistent or recurrent monoarthritis with an MRI before attributing a diagnosis of JIA [4, 9, 23–26]. Plain radiographs are of limited value, as they are normal in at least half of the patients. On ultrasonography, the IAVM is seen as an iso- or hyperechogenic mass with a posterior reinforcement, accompanied with enhanced venous vascularization [27]. Abnormal venous drainage around the joint can also be detected [15]. This technique may allow the definition of the size and location of the lesion, but images may be misleading and misinterpreted, as in our first case, as prominent synovial hypertrophy. Phleboliths, when present, are seen as brightly echogenic foci within the lesion [23].

Angiography and computed tomography (CT) scan are generally not necessary if MRI is employed [23, 26]. Indeed, as shown in our patients, the nature and extent of the lesion is more accurately judged by MRI. The mass appears isointense-to-hypointense in comparison to the muscle on T1-weighted images, and hyperintense on T2-weighted images, with a grade of heterogeneity related to recurrent intralesional thrombotic or hemorrhagic episodes. In comparison with fat, the lesions tend to appear much brighter on T2-weighted images. Contrast enhancement largely depends on the gradient of lymphatic or vascular composition [9, 24, 26].

As highlighted by Kan et al. [28], certain synovial lesions, such as IAVM, synovial sarcoma, and pigmented villonodular synovitis, display peculiar MRI features that may facilitate their distinction from other conditions containing blood products. In particular, IAVM can be diagnosed by identifying tubular structures within both the intra- and extra-articular spaces, and often extending to the extra-articular soft tissue and bone structures. These lesions may lack extra-articular edema [9, 28]. The degree of contrast enhancement may help to differentiate IAVM from cystic synovial hyperplasia [29].

IAVM can extend to fat tissue and muscles, but very rarely invade the bone [1, 30]. Intra-capsular and intra-synovial lesions are associated with a higher risk of chondral damage [16], but the size seems not to correlate with the risk of erosive arthropathy [31]. In our patients, the lesions were located in different knee areas, but all involved the synovium.

Although MRI may precise well the size of the mass, it may not be entirely accurate, and sometimes the exact extension can be defined only at surgery [9, 15].

Diagnostic arthroscopy can be helpful to confirm the nature of the lesion through the histopathologic assessment, as happened in our cases. However, nowadays arthroscopy is generally indicated only for cases in which the imaging specialist is not certain regarding the MRI findings, or if the findings are atypical. MRI is considered the modality of choice to make the diagnosis.

The histopathologic features of IAVM are in line with their definition as vascular malformations [8]. At microscopy, a synovium infiltrate composed by a mixture of medium-sized and large-sized vessels with a muscular wall of variable thickness, and thin-walled medium-sized vascular spaces is observed [1, 3].

Resection of knee IAVM by open surgery is considered the treatment of choice, as it can lead to long-term improvement of pain and joint mobility [3, 15]. In addition, the complete removal of the lesion is fundamental in order to avoid recurrence of intra-articular bleeding [3, 15, 17, 18]. It is unclear if treating asymptomatic patients can prevent chondropathy [15, 18, 32]. None our patients experienced recurrence of bleeding after surgery, even after many years. Another proposed treatment is based on sclerotherapy. This procedure may be helpful as a bridge therapy before open surgery or in case of extensive intra-articular lesions, but it is most frequently applied in soft tissue venous malformations. Sclerotherapy may reduce pain, but its effect is often transient [15, 18, 33].

Radiotherapy has been proposed in the management of IAVM, but is not used routinely. It might have a role in diffuse IAVM, when complete surgical removal is difficult and the growth plates are closed [13, 17].

In summary, our report highlights the challenges that may be posed by the detection of knee IAVM and the frequent long delay between the onset of symptoms and the correct diagnosis. A careful assessment of patient history, the demonstration of a bloody fluid upon arthrocentesis, and the proper interpretation of the MRI scanning may facilitate the recognition and early treatment of this lesion, and avoid the need for a diagnostic arthroscopy before surgical removal.

## Data Availability

Not applicable.

## Abbreviations

IAVM: Intra-articular venous malformation
JIA: Juvenile idiopathic arthritis
MRI: Magnetic resonance imaging
NSAID: Nonsteroidal antiinflammatory drug
PVNS: Pigmented villonodular synovitis
CT: Computed tomography
ISSVA: International Society for the Study of Vascular Anomalies
